# Viral Genetic Linkage Analysis in the Presence of Missing Data

**DOI:** 10.1371/journal.pone.0135469

**Published:** 2015-08-24

**Authors:** Shelley H. Liu, Gabriel Erion, Vladimir Novitsky, Victor De Gruttola

**Affiliations:** 1 Department of Biostatistics, Harvard School of Public Health, Boston, Massachusetts, United States of America; 2 School of Engineering and Applied Sciences, Harvard University, Cambridge, Massachusetts, United States of America; 3 Department of Immunology and Infectious Diseases, Harvard School of Public Health, Boston, Massachusetts, United States of America; Centro de Biología Molecular Severo Ochoa (CSIC-UAM), SPAIN

## Abstract

Analyses of viral genetic linkage can provide insight into HIV transmission dynamics and the impact of prevention interventions. For example, such analyses have the potential to determine whether recently-infected individuals have acquired viruses circulating within or outside a given community. In addition, they have the potential to identify characteristics of chronically infected individuals that make their viruses likely to cluster with others circulating within a community. Such clustering can be related to the potential of such individuals to contribute to the spread of the virus, either directly through transmission to their partners or indirectly through further spread of HIV from those partners. Assessment of the extent to which individual (incident or prevalent) viruses are clustered within a community will be biased if only a subset of subjects are observed, especially if that subset is not representative of the entire HIV infected population. To address this concern, we develop a multiple imputation framework in which missing sequences are imputed based on a model for the diversification of viral genomes. The imputation method decreases the bias in clustering that arises from informative missingness. Data from a household survey conducted in a village in Botswana are used to illustrate these methods. We demonstrate that the multiple imputation approach reduces bias in the overall proportion of clustering due to the presence of missing observations.

## Introduction

Targeting HIV prevention interventions to high-risk groups [1] can be aided by analyses of HIV viral genetic linkage. In particular, investigation of viral genetic sequences of study participants in community-based studies can reveal the viral strains propagating within and across communities and the characteristics of people infected with these strains. Numerous studies have already demonstrated the use of viral sequences from HIV-infected participants to investigate patterns of phylogenetic linkage, which provides information about patterns of HIV transmission dynamics [2–22].

In this paper, we consider data collected from a household survey in a village in Botswana that was done in conjunction with a pilot study of combination HIV prevention. Nonresponse is a major concern in these types of studies; contributing factors include work patterns, such as that of migrant workers and other highly mobile individuals, temporary absence of the individuals, and migration [23]. Because certain demographic groups, especially males, are systematically under-represented in the sample, methods to control for bias from missing data are required to make valid inferences about genetic linkage. Missing data may arise by happenstance or by design; consideration of the potential for bias in estimation of probabilities of linkage is especially important when the pattern of missingness may be related to characteristics of observed and missing observations—a pattern is that is referred to as informative missingness [24, 25]. We propose methods to adjust for the presence of informatively missing data in viral genetic linkage analysis.

We investigate viral linkage though examination of clustering, which is a measure of relatedness among viruses sampled from different people. One metric of interest is the overall proportion of clustering in the community, i.e., the proportion of prevalent HIV cases that are phylogenetically linked to other residents in the same community. In this study our definition of clustering is based on the pairwise distance between viral sequences; we define two individuals to be linked when this distance is below a specified threshold. Test statistics can help determine whether certain demographic groups are more likely to be clustered, which aids in understanding HIV transmission patterns—especially if sampled viral sequences include new infections. Linkage analyses can also reveal the overall proportion of clustering in the dataset, which provides information about the extent to which individuals are being infected by strains circulating inside their community or by strains outside of the community. Information regarding whether HIV transmissions are mainly occurring within or between communities can help reveal virus transmission dynamics, as well as identify subgroups of the population that are driving the epidemic [4, 6, 8, 13–17, 22, 26–30]. In the setting where an intervention is being provided, changes in the overall proportion of clustering within a village receiving the intervention over time are informative regarding the efficacy of the interventions; if it is working, there should be a decrease in virologically linked cases over time.

As mentioned above, a major problem arises in linkage analyses when the sample of viral sequences is incomplete; this situation may occur because intended subjects cannot be found or refuse participation. Bias arises even if observations are missing completely at random. Because viral linkage is based on the clustering of pairs of individuals, random missing links in the viral transmission chain will cause underestimation in the proportion of clustering. Since observing a link requires the presence of both linked sequences, removal of a subject removes all links to that subject. Hence, the number of missing links between sequences can increase more rapidly than does the number of missing subjects. Therefore, unbiased estimation of the true proportion of sequences that cluster requires information about both the total population size (the number of missing subjects plus the number of observed sequences), and characteristics that impact the probability that a sequence is missing.

When observations are informatively missing (i.e. viral genetic information is more likely to be missing for individuals of certain demographic groups), additional sources of bias are introduced. The fact that the presence of linkage depends on each sequence in the database creates a challenge for proper handling of missing data. Adjustment for missing data can be at the test statistic level [31] or at the viral sequence level. Carnegie et al. 2014 showed that the probability that individual sequences are linked can be consistently estimated using the observed data, and the probability that groups of sequences are linked can be estimated as well under the assumption that pairs of sequences are uncorrelated. The authors also developed a resampling approach for estimating the proportions of linkage that accounts for correlation between pairs of individuals. Here, we study a similar problem but use a multiple imputation approach framework that imputes missing sequences at the translated amino acid level, which is applicable in a very broad array of analyses that require imputed sequences, including phylogenetic analyses. In our investigation we focus on translated amino acid sequences as we are interested in non-synonymous mutations; previous research has shown that almost all site mutations within protein-coding regions are non-synonymous mutations [32]. Our focus here is on the use of imputed-complete datasets to investigate the proportion of overall HIV clustering in the targeted population. This paper investigates sensitivity of estimation for incomplete data, and the extent to which the proposed multiple imputation approach reduces bias in viral linkage estimates.

## Materials and Methods

The data we consider arises from a pilot study that provided information to aid in the design of a combination prevention cluster-randomized trial in Botswana [33]. The treatment modalities under investigation include testing of all consenting village subjects, provision of treatment for patients with viral loads above 10,000 copies/mL, and male circumcision for all HIV-uninfected men. The pilot study was undertaken in Mochudi, Botswana in 2011-2013, and provided household surveys, HIV testing for all consenting household members not previously diagnosed with HIV infection, laboratory assessment of viral load and CD4 lymphocyte count for HIV+ participants, and ascertainment of antiretroviral treatment status. The study was conducted according to the principles expressed in the Declaration of Helsinki, and was approved by the Health Research and Development Committee (HRDC) of the Republic of Botswana, and the Office of Human Research Administration (OHRA) of the Harvard School of Public Health. All adult study subjects provided written informed consent for participation in the study; all minor study subjects provided written informed assent, and each minor’s guardian provided written informed consent for their participation in the study. All available data were used for this paper; the 371 HIV-1 sequences analyzed in this paper are from patients enrolled in the first phase of the pilot study that was completed in Spring of 2012. HIV-1 subtyping revealed that 370 sequences were subtype C, and one sequence was HIV-1 subtype A1. Of the 371 subjects in the data, 287 were female and 84 were male, among whom 124 females and 24 males were categorized as young (<35 years). Young females accounted for 43% of females and young males accounted for 29% of males. For all virological samples, the V1C5 codon-based alignment was generated as described elsewhere [26] using muscle [34] in MEGA5 [35].

An estimate of the overall proportion of clustering, or the proportion of sequences that link to at least one other sequence, was calculated using the pairwise distance matrix of translated amino acid residues from phylogenetic analysis, which quantifies the degree of similarity between any two sequences. Several methods exist for calculating the distance matrix. These include Jukes-Cantor [36], Dayhoff [37], and JTT [38], among others. These methods assume different relationships among transition probabilities and different equilibrium frequencies. For simplicity of illustration, we define pairwise distance as the number of absolute amino acid changes between any two sequences divided by the total sequence length, but the viral sequence imputation method would work for other distance measures. *D*_*i*_*j*__ represents the pairwise distance between individuals *i* and *j*, and *N* is the total number of individuals in the dataset. If *D*_*i*_*j*__ is less than the threshold, then individuals *i* and *j* are considered to be clustered. Moreover, if *D*_*i*_*j*__ is less than the threshold for at least one individual j, where *i* ≠ *j*, then individual *i* is considered to cluster. We represent this by introducing an indicator variable, *I*_*i*_, for individual *i* such that:

$$\begin{array}{c} I_{i}=\{\begin{array}{cc} 1 & \text{if}\;D_{i_{j}}\;\text{is}\;\text{less}\;\text{than}\;\text{the}\;\text{threshold}\;\text{for}\;\text{at}\;\text{least}\;\text{one}\;\text{individual}\;\text{j,}\;\text{where}\;i\neq j\\ 0 & \text{Otherwise} \end{array} \end{array}$$

Overall proportion of clustering: $\frac{1}{N}\sum_{i=1}^{N}I_{i}$

We consider two thresholds for illustration of the sequence imputation method: 0.10 and 0.15. These thresholds were chosen based on the distribution of the V1C5 pairwise distances of the translated amino acids. Our methods apply for any threshold; appropriate choice of a clustering threshold is an active research area and can be affected by many factors, such as sampling density [39].

Our sequence imputation approach follows the multiple imputation statistical paradigm for dealing with informative missing data [24, 25]. Multiple imputation is a statistical method that allows for valid inferences from incomplete data, by creating imputed-complete datasets. We use the following formulas to calculate the mean proportion of clustering across imputed-complete datasets, the variance, standard deviation and standard error of the mean.

Following the notation of Rubin 1996, the *m* imputed-complete datasets have corresponding *m* estimated statistics [*Q*_.1_, …, *Q*_.*m*_] and variance-covariance matrices [*U*.1, …, *U*.*m*]. The repeated-imputation estimate is $Q_{m}=\sum_{l=1}^{m}Q_{.l}/m$, the associated variance-covariance of *Q*_*m*_ is *T*_*m*_ = *U*_*m*_ + (*m* + 1)/*mB*_*m*_, where $U_{m}=\sum_{l=1}^{m}U_{.l}/m$ is the within-imputation variability, which is calculated by bootstrap as described in [40]. A collection of bootstrapped alignments is generated by resampling the sites of the imputed alignment with replacement. Each bootstrapped alignment has the same number of sequences as the imputed alignment, but the column of nucleotides representing a given site in the imputed alignment may be present multiple times or not at all in the bootstrapped alignment. $B_{m}=1/(m-1)\sum_{I=1}^{m}(Q_{.I}-Q_{m}){\left(Q_{.I}-Q_{m}\right)}^{\prime }$ is the between-imputation variability.

**Fig 1:**
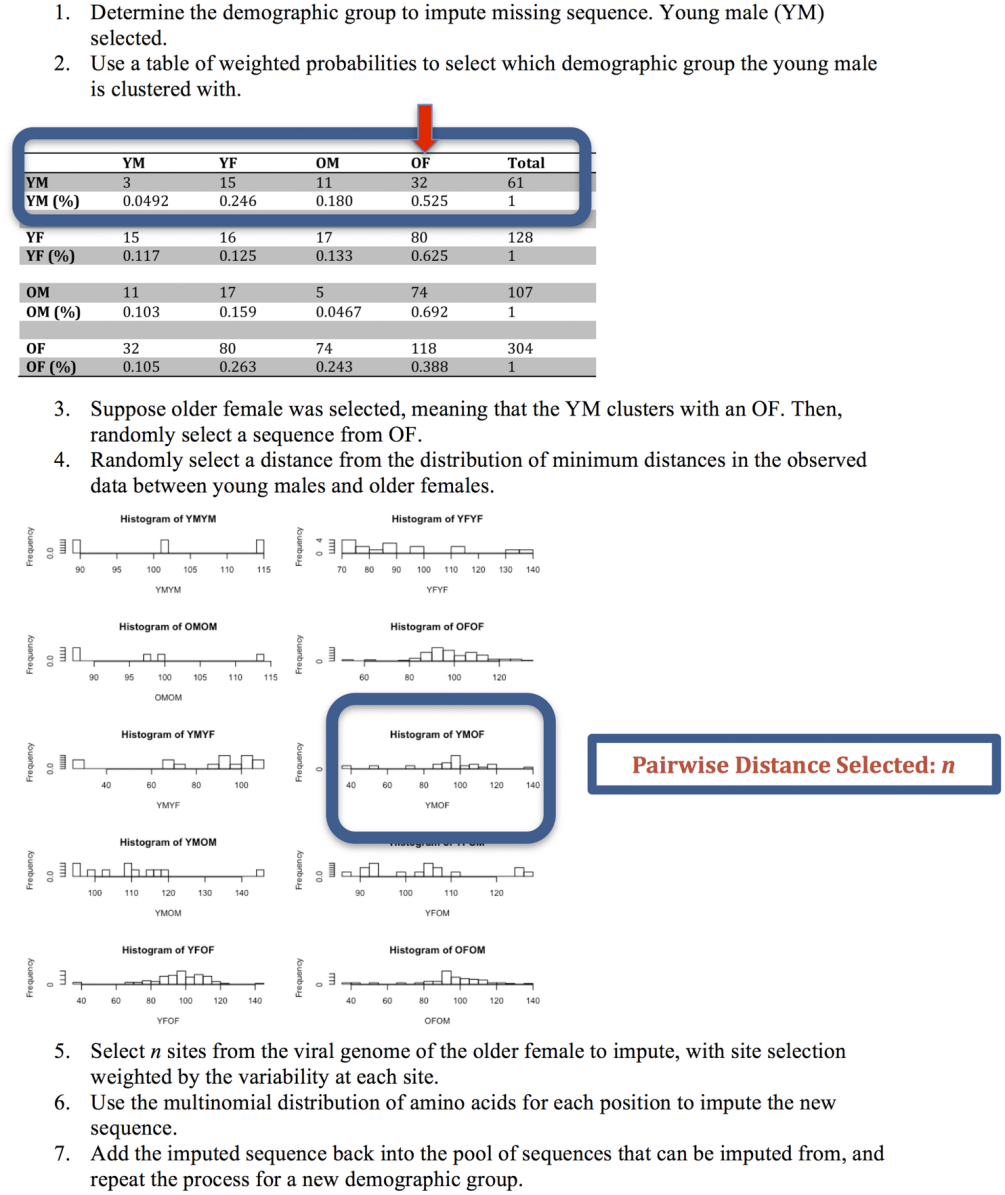
Schematic of the sequence-imputation method.

The following algorithm illustrates the sequence imputation mechanism, which is illustrated in Fig 1. Here we assume that demographic categorization is possible for everyone in the population, regardless of whether a sequence is available. *(1)* For each missing sequence, determine the demographic group, of which there are four: young males, young females, older males or older females. *(2)* Use a table of weighted probabilities to select the demographic group with which the subject whose sequences is missing is clustered. The weighted probabilities are the probabilities, calculated using observed sequences, that a sequence in one demographic group has its nearest neighbor in another demographic group. These probabilities are defined by the distribution of minimum distances, which is the minimum number of absolute amino acid changes from one sequence to the next. *(3)* Randomly select a viral sequence from this demographic group. *(4)* From the distribution of minimum distances in the observed data between the demographic groups of a subject with a missing sequence and the randomly selected sequence, select a distance, *n*, at random. This distance is the number of absolute amino acid changes between two sequences. *(5)* Select *n* sites from the viral genome of the sequence in *(3)* to modify. Site selection is weighted by the variability of each site, based on the marginal frequency of the most common amino acid at each site. Marginal frequencies for each amino acid site were computed using the Biopython package, which was also used for alignment parsing [41]. *(6)* Impute the amino acids for each of the *n* positions based on assumption that these amino acids are multi-nomially distributed. *(7)* Add the imputed sequence back into the pool of sequences that can be imputed from.

## Applications

### Simulation Study

A simulation study demonstrated the ability of the model to reduce the biases arising from informative missing data. The study was based on incomplete datasets that were created by deleting sequences from the available data, and then using the multiple imputation procedure to estimate rates of clustering for the entire observed database of 371 sequences. Deletions of *m* = (10, 20, …, 200) subjects were made. We refer to the sample of *N* = 371 sequences as the observed data set; the observed data with *m* deletions that yield *N* = 371 − *m* sequences as the incomplete-observed data; and the incomplete-observed dataset plus the imputed values as the imputed-complete data.

**Fig 2:**
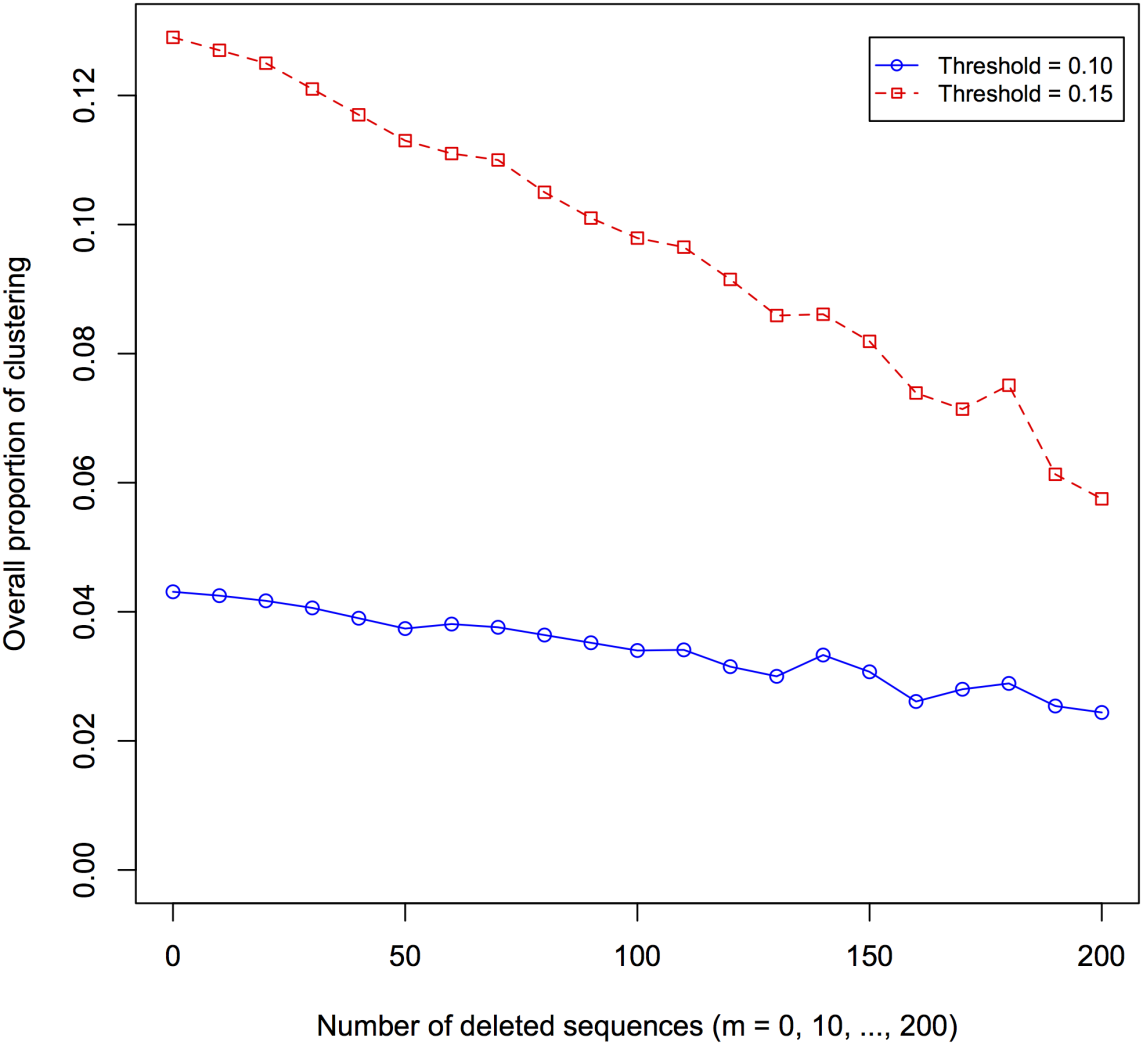
Decrease in overall proportion of clustering with deletion of sequences. Clustering at each number of deletions is averaged over 100 different random deletions of the same number of sequences.

Fig 2 demonstrates that as the number of deleted sequences increases, the proportion of overall clustering decreases. The sequences were deleted selectively; weighting was by gender, with sequences of females deleted ten times more often than that of males. Because of the dearth of young males in the observed dataset, the male:female deletion ratio was chosen to preserve sequences of young males in the incomplete-observed datasets when *m* was large. The proportion of subjects who cluster with others is naturally larger with a higher threshold (0.15 instead of 0.10).

Table 1 shows that the adjustment using multiple imputation reduces—and in some cases nearly eliminates—the bias resulting from missing data. We consider incomplete-observed datasets created by deleting *m* = 50, 100, 200 sequences from the observed dataset and calculate the mean proportion of clustering for the thresholds of 0.10 and 0.15. The variance was calculated as described in the Methods section. The table also provides the coverage percentages for the 95% confidence intervals on estimates from deletion and imputation. The confidence intervals were obtained by bootstrapping standard errors. For each incomplete-observed dataset, in which *m* sequences had been deleted, ten imputed-complete datasets were constructed to calculate the imputed-complete estimates and standard errors. This process was repeated over 100 different subset deletions (incomplete datasets with *m* deletions) in order to calculate coverage. Coverage was defined as the proportion of times that 100 repetitions over 100 different subset deletions yielded a 95% confidence interval that included the clustering value obtained from the complete data. As the amount of missing data increases, the performance of the multiple imputation estimates and the coverage worsens, but the bias is always considerably decreased and coverage improved compared to the results for the incomplete-observed data.

**Table 1:** Estimated overall proportion of clustering for observed, incomplete-observed and imputed-complete datasets.

Thres	Clus, Obs	# Del	Clus, In-Obs	Clus, Impute-Com	Cov, In-Obs	Cov, Impute-Com
0.1	0.043	50	0.039	0.043	83%	94%
		100	0.036	0.043	66%	89%
		200	0.024	0.037	30%	61%
0.15	0.13	50	0.11	0.13	48%	91%
		100	0.099	0.13	16%	85%
		200	0.059	0.10	1.4%	54%

*Thres* stands for threshold; *Clus* for clustering; *Obs* for observed data; *# Del* for number of deleted sequences; *In-Obs* for ncomplete-observed data; *Impute-Com* for imputed-complete data; *Cov* stands for coverage. Clustering assessed at the 0.10 and 0.15 thresholds, with varying number of deleted sequences (m = 50, 100, or 200).

**Fig 3:**
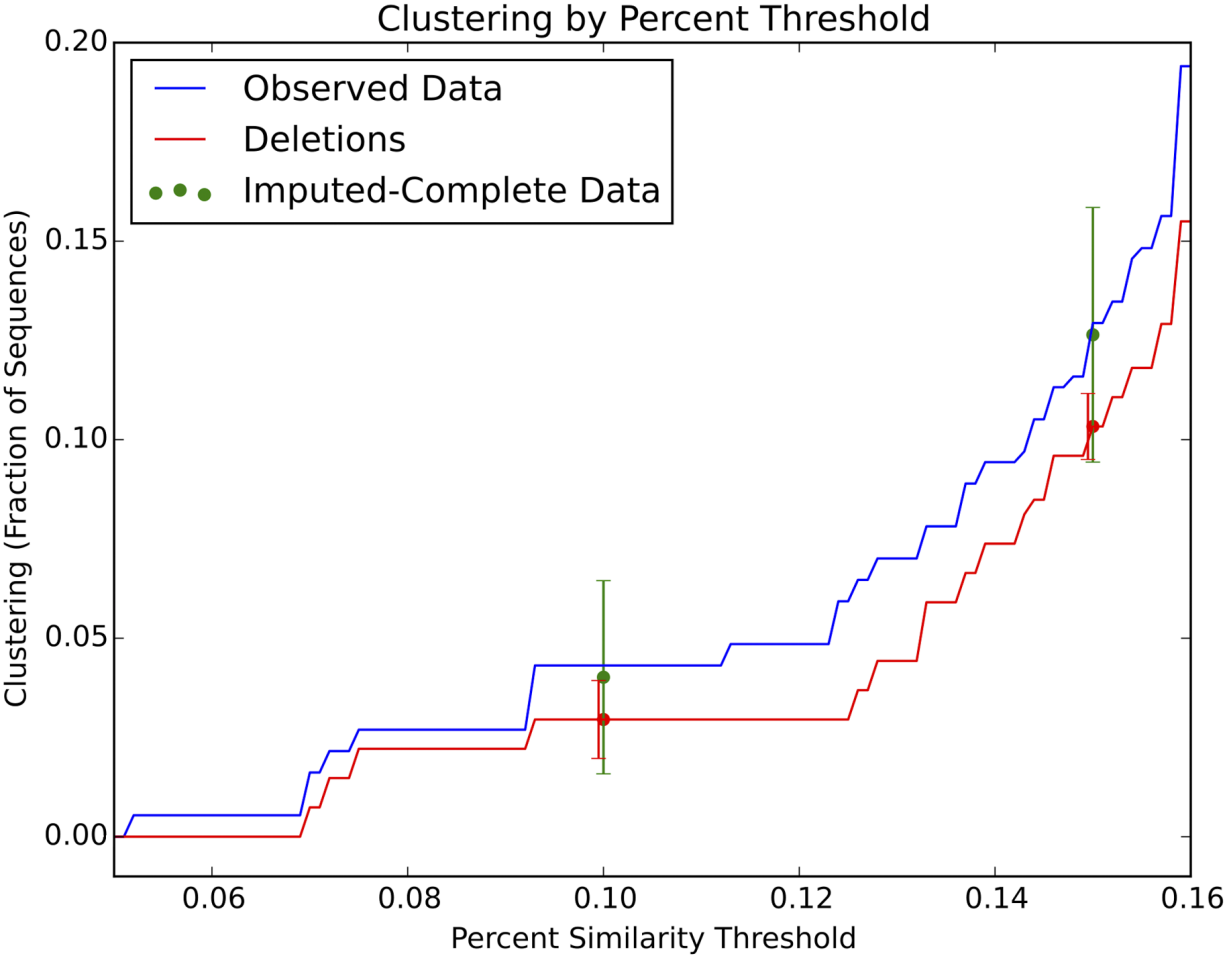
Demonstrating the corrective effect of a representative imputation. Deleting 100 sequences leads to noticeably lower clustering. Imputation substantially improves estimates, and results in slightly larger error bars.

Fig 3 demonstrates the bias-correcting effect of the multiple imputation. Deleting 100 sequences leads to noticeably lower clustering. For the incomplete-dataset, at the 0.10 threshold, overall proportion of clustering is 0.030 [0.020, 0.039]; at the 0.15 threshold, overall proportion of clustering is 0.10 [0.095, 0.11]. The multiple imputation method substantially reduces bias and results in larger 95% confidence intervals. For the imputed-complete dataset, at the 0.10 threshold, the overall proportion of clustering is 0.040 [0.016, 0.064]; at the 0.15 threshold, the overall proportion of clustering is 0.13 [0.094, 0.16].

We investigate the performance of the sequence imputation method for two scenarios for sampling data missing not at random (MNAR) in Table 2. For the first, sequences are deleted from the observed-complete dataset in proportion to the number of other sequences with which they cluster, plus one. If, for example, a sequence clusters with 9 other sequences, it will be ten times more likely to be deleted than a sequence which did not cluster with any other sequences. Under the second scenario, sequences are deleted in a manner inversely proportional to their number of clustering partners. For each incomplete-observed dataset, in which *m* = 100 sequences were deleted, ten imputed-complete datasets were constructed to calculate the imputed-complete estimates and standard errors. This process was repeated over 1000 different subset deletions (incomplete datasets with *m* = 100 deletions) in order to calculate coverage. The results demonstrate that serious violation of assumptions regarding the nature of the missingness process greatly impact results. As expected, clustering estimates from the imputed-complete dataset under proportional deletions are much smaller than the analogous estimates under inversely proportional deletions.

**Table 2:** Estimated overall proportion of clustering for observed, incomplete-observed and imputed-complete datasets under MNAR.

Deletion weight	Clus, Obs	Clus, In-Obs	Clus, Impute-Com	Cov, In-Obs	Cov, Impute-Com
Proportional	0.043	0.018	0.022	8%	42%
Inversely proportional		0.043	0.053	80%	93%

Clustering assessed at the 0.10 threshold, with *m* = 100 deletions. *Clus* stands for clustering; *Obs* for observed data; *In-Obs* for ncomplete-observed data; *Impute-Com* for imputed-complete data; *Cov* stands for coverage.

### Mochudi Pilot Study

We applied our methods to adjust for the fact that the observed Mochudi pilot dataset is an incomplete, biased sample of the population of interest. The focus of estimation is the true proportion of the clustering in the entire population targeted by the Mochudi pilot study. To improve estimation, we impute missing sequences to create an imputed-population dataset. Although we do not have exact information on the number of missing observations, we do have information about the age-gender structure for Botswana as a whole as well as information about HIV-1 prevalence by age and gender in Mochudi. While we cannot be certain that the age-gender structure for Mochudi resembles that of Botswana as a whole, the former is likely to be much closer to the structure for the country than is the observed sample from Mochudi, given its notable under-representation of males. The 2013 CIA World Factbook provides an age-gender breakdown for Botswana [42], which we divide into four categories: young males/females (15-35 years) and older males/females (> 35 years). In order to obtain the estimated proportion of HIV-1 infected people in each age-gender category in Mochudi, we multiply the proportion of people in each age-gender category for Botswana as a whole by the estimated prevalence in those categories, obtained from the Mochudi pilot study [43]. From these calculations we can estimate the proportions of HIV-infected subjects that are in each age-category in Mochudi. We then add imputed sequences to our observed dataset so that the proportions of subjects in each age-gender category of our augmented sample matches those based on our estimates. The estimated proportions are: young males (0.097), older males (0.292), young females (0.295), older females (0.317). To achieve these proportions, we impute sequences for 84 older males, 0 older females, 24 young males, and 25 young females; a total of 133 sequences are added to our observed dataset of 371 sequences. We note that while this database may not be complete, it should be considerably closer to complete than is the observed sample, as older women were most likely to participate in the Mochudi pilot [43]. Furthermore, the imputation adjusts for the under-sampling of males.

**Fig 4:**
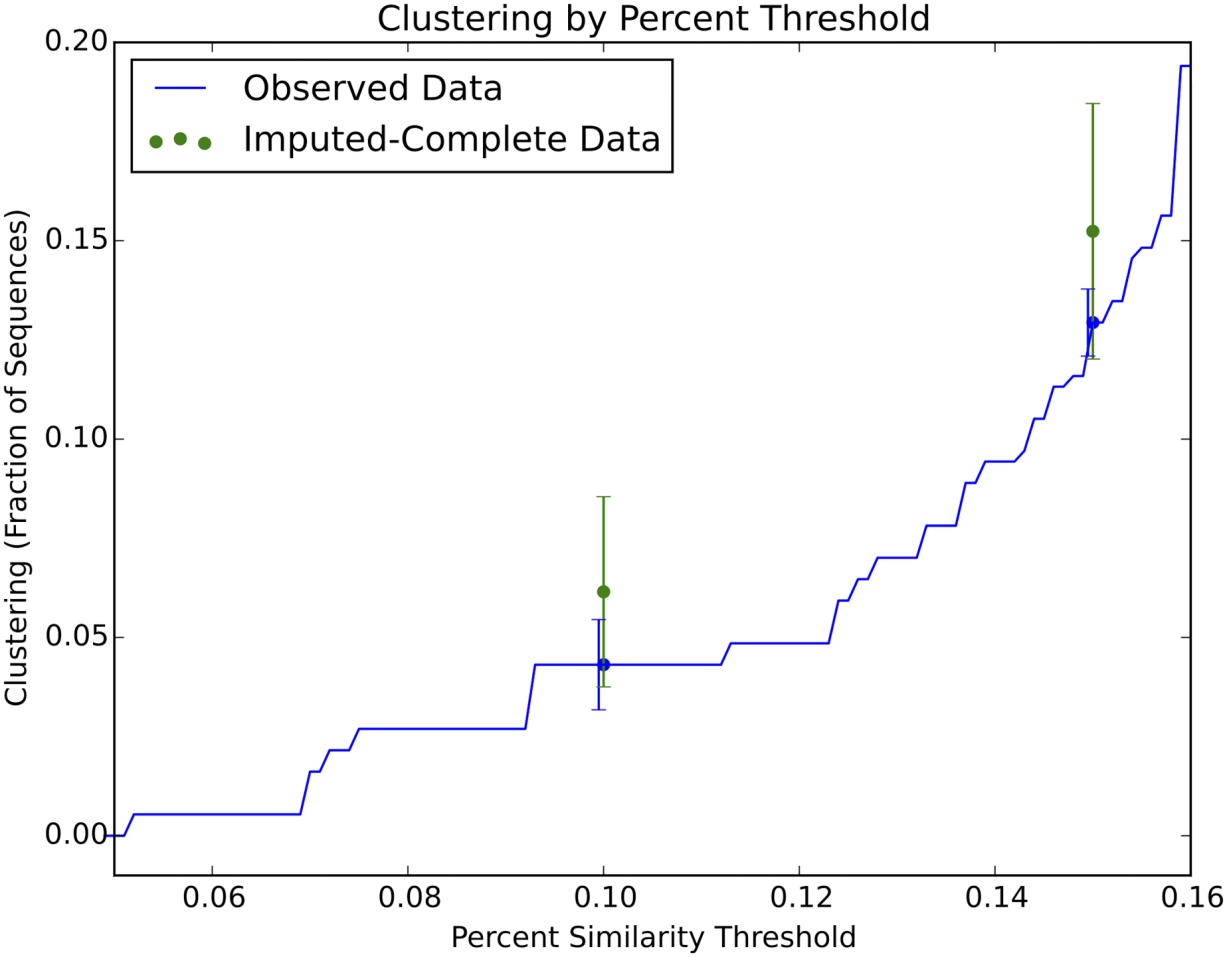
Estimating the true proportion of clustering in the Mochudi population. Treating the observed Mochudi data (N = 371) as a biased sample from the population, and imputing the imputed-population dataset (N = 504) based on Botswana and Mochudi-specific age and gender breakdowns to create a database with the same demographic structure as Botswana as a whole. Point estimates and error bars for the 0.10 and 0.15 thresholds of clustering.

Fig 4 shows the effect of applying our viral sequence imputation method to the observed sequence data. In the observed sample (N = 371), the proportion of overall clustering was estimated to be 0.043 [0.034, 0.053] at the 0.10 clustering threshold and 0.13 [0.11, 0.14] at the 0.15 clustering threshold. In the imputed-population dataset (N = 504), the proportions of overall clustering at the two thresholds were 0.062 [0.038, 0.086] and 0.15 [0.12, 0.18], respectively. We note that for both the 0.10 and 0.15 thresholds, the upper confidence interval for the clustering in the observed data excludes the point estimate for the imputed-population dataset. The clustering estimates for the imputed-population data reflect a lower bound as there are likely more missing sequences than the 133 we imputed; if some older females are missing, then additional imputed sequences in all categories would be required.

## Discussion

To assess clustering and viral linkage in the presence of missing data, we propose an approach that combines a traditional missing data framework with a model for viral diversification after transmission. In the setting we consider, our sequence imputation method corrects for biases in viral genetic linkage analyses in the presence of informatively missing data. The performance of the multiple imputation approach appears to depend on the proportion of data that are missing. We implemented simulation studies in which our procedures are used to make inferences on the incomplete-observed dataset, and compared the results with those from the complete-observed dataset.

In our simulation study, the proposed method performed well when the proportion missing is less than 30%, but underestimated the proportion of sequences that are clustered when the proportion missing is over 50%. One possible reason for this bias is in the distribution of minimum pairwise distances, from which we randomly select the number of amino acid changes to impute from the selected sequence. Bias arises from the fact that this distribution is based on observed distances from an incomplete sample. The distribution of minimum distances is increasingly shifted to the right with greater number of deletions because of the smaller number of links in the incomplete data. To investigate this issue, we conducted a sensitivity analysis as follows: we used the distribution of minimum distances from the full observed dataset rather than from the incomplete dataset in the sequence imputation approach. This approach, however, did not significantly reduce the bias. Next, we investigated the sensitivity to use of a kernel density estimator for estimating the distribution of minimum distances, compared to using the ad-hoc method. The results in S1 Table do not suggest any major difference in results when using kernel density estimation compared to the approach described above. We also conducted a sensitivity analysis to determine if the proportions of sequences deleted by gender affected the imputed-complete clustering estimates in our simulation study. We simulated male:female sequence deletion ratios ranging from 1:1 to 1:10 (M:F); the latter was the ratio used in simulation studies throughout the paper. S2 Table does not show a substantial impact of the deletion ratios on the performance of our method. Bias may also arise from limitations in the model for viral diversification—future studies based on clonal rather than population sequences may provide more information for this model. The lower coverage noted when the proportion of data that is missing exceeds 50% likely reflects not only lower amount of information used to estimate the quantities of importance (distances between genotypes), but also more reliance on generating sequences that may be several transmissions away from someone with an observed sequence.

To impute sequences requires a number of assumptions. One regards the covariation between amino acid positions of the *gp120* region; we assumed independence, but other choices are possible. A second assumption is that the clustered sequences have similar processes of diversification as those of the sequences of the larger population from which these clustered sequences are sampled. In addition, we assume that sequences are missing at random given observed characteristics on the missing sequences (e.g. age and sex of the hosts).

In this paper, we do not provide detailed guidance on choice of clustering threshold. The sequence imputation method is applicable to a diverse range of distance matrix definitions, and we present a simple distance matrix definition for ease of illustration. In practice, thresholds will be specific both to the distance matrix definition and the study objective. For example, in assessing whether two individuals are likely to be fairly closely connected in the same transmission chain, it might be preferable to use a lower threshold. By contrast, assessing whether two individuals are infected by strains circulating within a given community or by strains circulating only outside of the community, one might prefer a higher threshold. We recommend investigation at a range of thresholds as shown above, although we selected two thresholds to demonstrate coverage.

In the simulated imputed-population dataset, in which we apply our method to adjust for the bias and incompleteness of the observed Mochudi pilot dataset, our estimates of clustering have limitations that arise from the sampling of the pilot study population in Mochudi [43]. We solely adjust for biases that result when the probability of sampling depends only on age category and gender. We note that we cannot exclude the possibility that probabilities of sampling depend on other factors as well. Nonetheless, we demonstrate that the sequence imputation method used to adjust clustering estimates can greatly improve estimation if these assumptions are met. The method could easily accommodate other factors that impact probability of sampling, when they are known and measured..

In summary, viral genetic linkage analyses has been shown to be useful in making inferences about transmission patterns, but analyses can yield biased results unless the impact of incomplete sampling of populations of interest is properly taken into account.

## Supporting Information

S1 Table Estimated overall proportion of clustering for observed, incomplete-observed and imputed-complete datasets using kernel density estimation for the distribution of minimum distances. Clustering assessed at the 0.10 threshold, with m = 100 deletions. Coverage is calculated as described in Table 2. Ratio of male:female deletions was 10:1. *Clus* stands for clustering; *Obs* for observed data; *In-Obs* for ncomplete-observed data; *Impute-Com* for imputed-complete data; *Cov* stands for coverage.
(PDF)

S2 Table Estimated overall proportion of clustering for observed, incomplete-observed and imputed-complete datasets at varying ratios of male:female sequence deletions. Clustering assessed at the 0.10 threshold, with m = 100 deletions. Coverage is calculated as described in Table 2. *Clus* stands for clustering; *Obs* for observed data; *In-Obs* for ncomplete-observed data; *Impute-Com* for imputed-complete data; *Cov* stands for coverage.
(PDF)
