# Supplementary material for: Viral Genetic Linkage Analysis in the Presence of Missing Data
Source: PLoS One. 2015 Aug 24;10(8):e0135469. doi: 10.1371/journal.pone.0135469 (PMC4547719; doi:10.1371/journal.pone.0135469)
Supplement: S1 Table — Clustering assessed at the 0.10 threshold, with m = 100 deletions. Coverage is calculated as described in Table 2. Ratio of male:female deletions was 10:1. Clus stands for clustering; Obs for observed data; In-Obs for ncomplete-observed data; Impute-Com for imputed-complete data; Cov stands for coverage. (PDF) [file pone.0135469.s001.pdf]

S1 Table. Estimated overall proportion of clustering for observed, incomplete-observed and imputed-complete datasets using kernel density estimation for the distribution of minimum distances

Approach	Clus, Obs	Clus, In-Obs	Clus, Impute-Com	Cov, In-Obs	Cov, Impute-Com
Ad-hoc	0.043	0.034	0.042	56%	88%
Kernel density		0.034	0.040	56%	91%

Clustering assessed at the 0.10 threshold, with m=100 deletions. Coverage is calculated as described in Table 2. Ratio of male:female deletions was 10:1. *Clus* stands for clustering; *Obs* for observed data; *In-Obs* for ncomplete-observed data; *Impute-Com* for imputed-complete data; *Cov* stands for coverage.
