# Supplementary material for: Viral Genetic Linkage Analysis in the Presence of Missing Data
Source: PLoS One. 2015 Aug 24;10(8):e0135469. doi: 10.1371/journal.pone.0135469 (PMC4547719; doi:10.1371/journal.pone.0135469)
Supplement: S2 Table — Clustering assessed at the 0.10 threshold, with m = 100 deletions. Coverage is calculated as described in Table 2. Clus stands for clustering; Obs for observed data; In-Obs for ncomplete-observed data; Impute-Com for imputed-complete data; Cov stands for coverage. (PDF) [file pone.0135469.s002.pdf]

S2 Table. Estimated overall proportion of clustering for observed, incomplete-observed and imputed-complete datasets at varying ratios of male:female sequence deletions

Ratio (M:F)	Clus, Obs	Clus, In-Obs	Clus, Impute-Com	Cov, In-Obs	Cov, Impute-Com
1:1		0.031	0.046	47%	87%
1:2	0.043	0.033	0.044	54%	89%
1:5		0.034	0.043	59%	90%
1:10		0.034	0.042	56%	88%

Clustering assessed at the 0.10 threshold, with m=100 deletions. Coverage is calculated as described in Table 2. *Clus* stands for clustering; *Obs* for observed data; *In-Obs* for ncomplete-observed data; *Impute-Com* for imputed-complete data; *Cov* stands for coverage.
